# Blood flow but not cannula positioning influences the efficacy of Veno-Venous ECMO therapy

**DOI:** 10.1038/s41598-022-23159-z

**Published:** 2022-12-05

**Authors:** Massimiliano Leoni, Johannes Szasz, Jens Meier, Luca Gerardo-Giorda

**Affiliations:** 1 Johann Radon Institute for Computational and Applied Mathematics, Linz, Austria; 2 Department of Anaesthesiology and Intensive Care, Kepler University Klinikum, Linz, Austria; 3 Institute for Mathematical Methods in Medicine and Data Based Modelling, Johannes Kepler University, Linz, Austria

**Keywords:** Computational science, Cardiology, Diseases

## Abstract

Despite being vital in treating intensive-care patients with lung failure, especially COVID-19 patients, Veno-Venous Extra-Corporeal Membrane Oxygenation does not exploit its full potential, leaving ample room for improvement. The objective of this study is to determine the effect of cannula positioning and blood flow on the efficacy of Veno-Venous Extra-Corporeal Membrane Oxygenation, in particular in relationship with blood recirculation. We performed 98 computer simulations of blood flow and oxygen diffusion in a computerized-tomography-segmented right atrium and venae cavae for different positions of the returning and draining cannulae and ECMO flows of 3 L/min and $5\,\hbox {L}/\hbox {min}$. For each configuration we measured how effective Veno-Venous Extra-Corporeal Membrane Oxygenation is at delivering oxygen to the right ventricle and thus to the systemic circulation. The main finding is that VV-ECMO efficacy is largely affected by the ECMO flow (global peak blood saturation: ${92.8}\,\%$; average inter-group saturation gain: 9 percentage points) but only scarcely by the positioning of the cannulae (mean saturation ± standard deviation for the 3 L/min case: $83.12 \pm 0.49$; for the $5\,\hbox {L}/\hbox {min}$ case: $92.21 \pm 0.37$). An important secondary outcome is that recirculation, more intense with a higher ECMO flow, is less detrimental to the procedure than previously thought. The efficacy of current ECMO procedures is intrinsically limited and fine-tuning the positions of the cannulae, risking infections, offers very little gain. Setting a higher ECMO flow offers the biggest benefit despite mildly increasing blood recirculation.

## Introduction

Severe Acute Respiratory-Distress Syndrome (ARDS) is a life-threatening complication of COVID-19 infection. Despite the capabilities of modern medicine, ARDS mortality is still high^1^. Intubation, controlled ventilation, neuromuscular blockade, inhaled nitric oxide and prone positioning show little effect on the course of these patients due to decreased lung compliance and impaired gas exchange, as previously observed in patients suffering from pneumonia, sepsis or trauma^2,3^.

Extra-Corporeal Membrane Oxygenation (ECMO) is an advanced therapy for these patients. There are two different types of ECMO therapy: one offers cardiorespiratory support (VA-ECMO) whereas the other one offers sole respiratory support (VV-ECMO). The VV-ECMO therapy offers oxygenation and decarboxylation of the blood, facilitates recovery of the lungs and prevents further damage caused by harmful positive pressure ventilation^4,5^.

VV-ECMO drains deoxygenated blood from the patient via an inserted cannula, oxygenates and decarboxylates the blood outside the body in an oxygenator and returns the oxygenated blood back into circulation. There are various cannulation strategies (VA vs VV, central vs peripheral, three vs two vs single site cannulation)^6,7^. The main goals of a VV-ECMO therapy in COVID-19 patients are to maximize the oxygen content while minimizing ventilatory efforts and to reduce recirculation in patients needing high blood flow^6,8,9^.

Recirculation is a phenomenon unique to the VV-ECMO therapy where oxygenated blood from the returning cannula flows directly into the draining cannula without delivering oxygen to the organs. The effectiveness of a VV-ECMO therapy is believed to be mainly impaired by recirculation and to be affected by the distance between the draining and the returning cannulae^10^.

The first attempt to stabilise a critically-desaturating patient on ECMO support is to increase the blood flow. Increased drainage can lead to an increased fraction of recirculation and less oxygenated blood reaching systemic circulation, with all known consequences^9^.

Despite the importance of estimating the recirculating fraction^9^

1 $$\begin{aligned} RF = \frac{SO_2^{preoxy} - S_V O_2}{SO_2^{postoxy} - S_V O_2}, \end{aligned}$$

there are no easy methods to quantify recirculation or the true $S_VO_2$ as a marker for it. Existing techniques include the Central Venous Line, which is inaccurate as it measures the $S_{CV}O_2$ in the lower part of the superior vena cava instead. The SVO2 approach proposes to turn the sweep gas off until $SO_2^{preoxy}$ equals $SO_2^{postoxy}$. This technique might harm critically-ill patients^9^.

Other methods measure recirculation via thermodilution or ultrasound dilution techniques^11,12^. Those findings are limited in their clinical use because they are based on animal data, fixed cannulation strategies or dual lumen cannulae. Recent approaches calculate recirculation using computational models^13,14^.

The aim of this study is to create a computational model based on thoracic CT-scans to investigate how the efficacy of VV-ECMO is affected by ECMO blood flow and the position of the cannulae in order to optimise the outcome of the procedure. We will pay special attention to recirculation.

## Methods

We simulated blood flow and oxygen diffusion in a real-patient geometry for different positions of the cannulae. We then evaluated the efficacy of each configuration in terms of oxygenation of the blood entering the right ventricle.

### Model

#### Patient geometry

**Figure 1:**
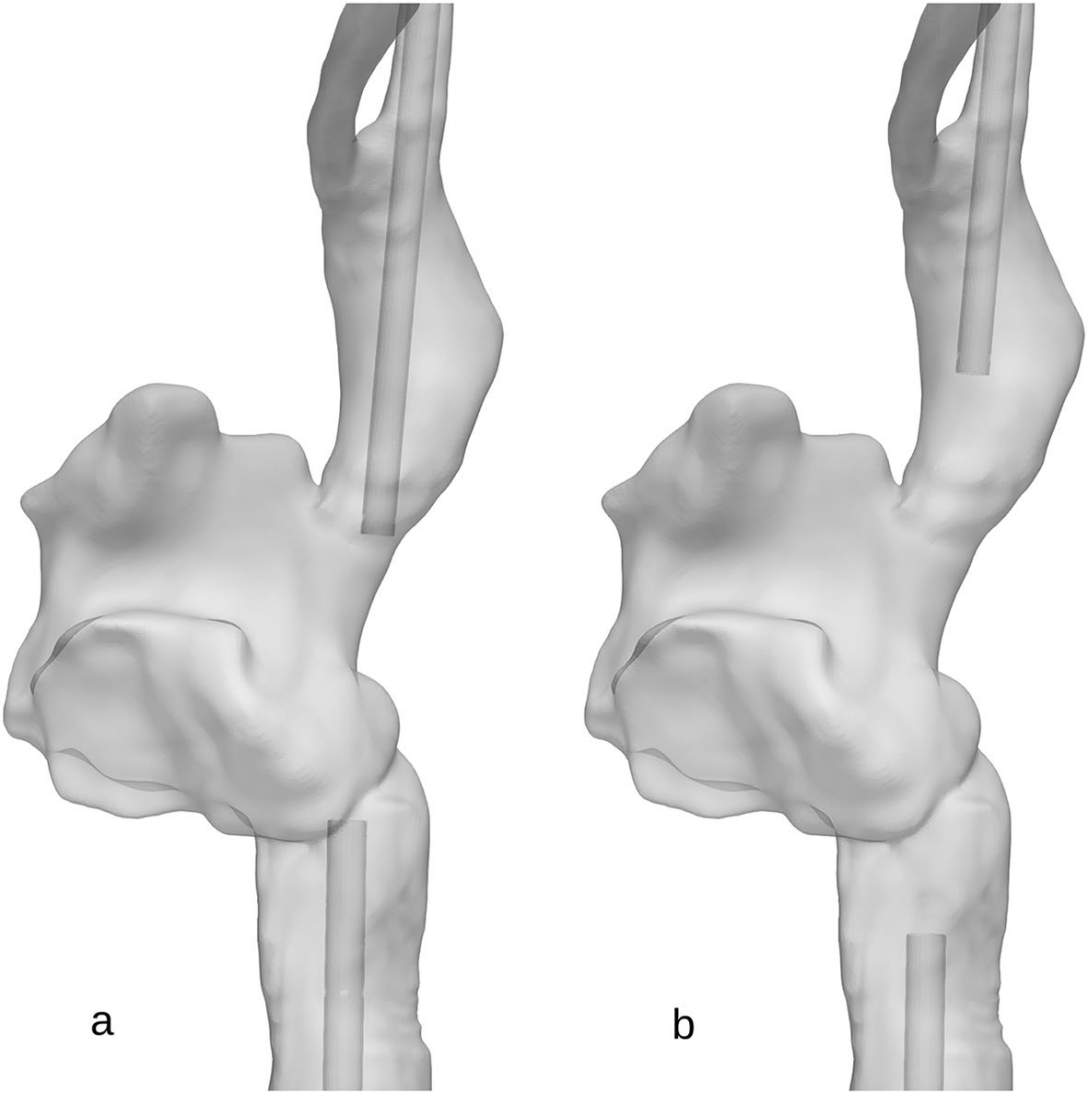
CT-segmented geometry for two configurations. Reconstructed patient anatomy with two different position configurations of the draining and returning cannula. Left: both cannulae are placed at the entrance to the right atrium; this configuration is labelled $(0,0)$. Right: The draining cannula is located at 2 cm from the atrium entrance, while the returning cannula is located at 3 cm from the entrance; this configuration is labelled $(2,3)$.

 We reconstructed portions of the superior and inferior venae cavae and the right atrium from Computer Tomography of a male ECMO patient obtained from Kepler University Clinic according to their approved guidelines. The patient provided a signed informed consent to authorise the use of his medical data in this study. The ethics committee of the medical faculty of the Johannes Kepler University in Linz evaluated this study and waived the requirement of an ethical approval. We included realistic models of the cannulae that are currently used in clinical practice (Fig. 1). Further details can be found in the additional files (see Additional file 1).

#### Mathematical formulation

 For the blood simulation we imposed conservation of momentum and mass with the incompressible Navier-Stokes equations. Oxygen diffusion is described by a standard advection-diffusion equation where the advection velocity is the blood velocity. Further details can be found in the additional files (see Additional file 1).

### Study set-up

#### Physiological parameters

 We consider two different ECMO flows, 3 L/min and $5\,\hbox {L}/\hbox {min}$, well within the typical 2–5 L/min range reported in the literature. The heart rate and the cardiac output are affected by the ECMO flow as described in^15^; unfortunately, no such information was available for the patient whose scans we used in our study. Thus, we correlated the cardiac output and the heart rate with the ECMO flow by linear regression on the data for the eight ARDS patients reported in^15^. The resulting setup values for our simulations are summarised in Table 1.

**Table 1:** The parameters used in our simulations setup.

Simulation parameters		
ECMO flow (L/min)	Heart rate (bpm)	Cardiac output (L/min)
3	134	5.76
5	81	3.81

#### Positioning

 Our base configuration, which we label $(0, 0)$, has both cannulae just outside the entrance to the right atrium. A configuration labelled, for example, $(n, p)$ means that the draining cannula is at $n \, \mathrm{cm}$ from the lower entrance to the right atrium and the returning cannula is at $p \, \mathrm{cm}$ from the upper entrance to the right atrium (see Fig. 1).

For each ECMO flow we study configurations with both cannulae at distances varying from 0 cm to 6 cm in steps of 1 cm, for a total of 49 configurations.

#### Simulation protocol

 The simulations use the finite elements method and run on a self-developed code based on the open-source software FEniCS-X. All cases ran for 64 s and data was recorded only in the last 30 s. Any incomplete heart beats in the time interval $34 - 64$ s were discarded.

### Effectiveness assessment and saturation

For each configuration we record all $N$ complete heartbeats in the final 30 s of the simulation. We denote by $F^k$ the amount of oxygen that actually flows into the right ventricle during the $k$-th heart beat and by $F^k_\text {max}$ and $F^k_\text {ven}$ the amounts of oxygen that would flow if the blood were either fully-saturated or purely venous. We evaluate the effectiveness of an ECMO configuration as the arithmetic mean over these $N$ heart beats

2 $$\begin{aligned} e = \frac{1}{N} \sum _{k=1}^N \frac{F^k - F^k_\text {ven}}{F^k_\text {max} - F^k_\text {ven}}. \end{aligned}$$

With this definition the effectiveness is a number between 0 and 1: it is 0 if only venous blood flows through the Tricuspid valve and it evaluates to 1 if as much oxygen as possible flows through the Tricuspid valve. In other words, this number indicates how much, of all the blood that goes through the Tricuspid valve, is fully saturated. Assuming a blood saturation in the veins at ${70}\,\%$^16^, we can estimate, for a given configuration, the average saturation at the Tricuspid valve as $S = 70 + 30e$.

## Results

### Saturation

**Figure 2:**
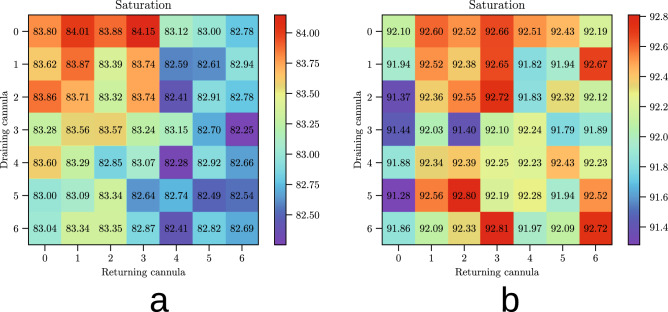
Saturation charts for the two different ECMO flows. Saturation levels at the Tricuspid valve for different cannula positions and the two considered ECMO flows. (**a**) 3 L/min. Mean: 83.12, standard deviation: 0.49. (**b**) $5\,\hbox {L}/\hbox {min}$. Mean: 92.21, standard deviation: 0.37. In both charts, the values span a small range, meaning that moving the cannulae influences the ECMO efficacy by less than ${2}\,\%$, hardly compensating the risk of generating an infection. On the other hand, the worst $5\,\hbox {L}/\hbox {min}$ configuration performs much better than the best 3 L/min one, indicating that increasing the ECMO flow offers the most benefit.

Figure 2 reports the computed saturation $S$ for the 98 configurations that we studied for ECMO flows of $5\,\hbox {L}/\hbox {min}$ and 3 L/min, respectively.

In Fig. 2 we can see that all the values in the saturation chart for the $5\,\hbox {L}/\hbox {min}$ case are in the interval $[91.2, 92.8]$, while the 3 L/min values span the $[82.2, 84.2]$ range.

Increasing the ECMO flow from 3 L/min to $5\,\hbox {L}/\hbox {min}$ gives an average gain in blood saturation ($S$) of around 10 percentage points regardless of the positions of the cannulae.

**Figure 3:**
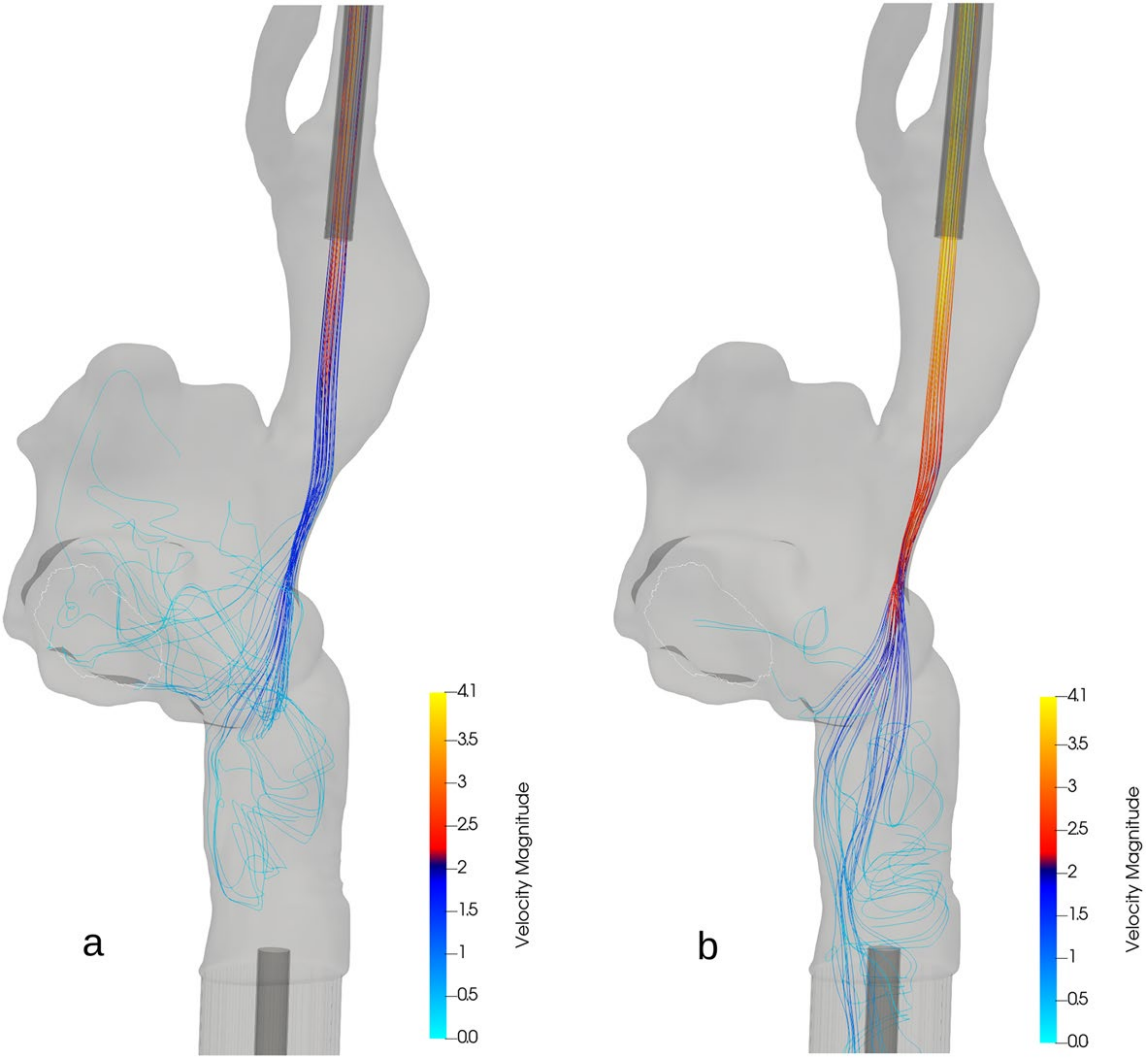
Streamlines for the blood flow for the same configuration with two different ECMO flows. Streamlines showing the path of the oxygenated blood from the returning cannula for the same configuration $(5, 5)$ with two different ECMO flows, coloured by the blood velocity. Both snapshots are taken while the Tricuspid valve is open. In the 3 L/min case the oxygenated blood, coming in at moderate speed, flows mostly into the Tricuspid valve. In the $5\,\hbox {L}/\hbox {min}$ case a bigger share of the injected blood, coming in much faster, flows through the right atrium and recirculates into the draining cannula. A video of the time evolution of the streamlines is available in Additional file 2.

### Flow dynamics and recirculation

Figure 3 compares, for the $(5, 5)$ configuration, the circulation patterns of the 3 L/min and $5\,\hbox {L}/\hbox {min}$ ECMO flows while the Tricuspid valve is open. The figure shows the streamlines of blood flow entering from the returning cannula, coloured by the blood’s velocity.

We see from inspection of Fig. 3 that the configuration with the smaller inflow distinguishes itself: in the 3 L/min case the majority of the blood coming in from the returning cannula flows into the Tricuspid valve, accounting for very little recirculation. In the $5\,\hbox {L}/\hbox {min}$ case, on the other hand, the high speed of the inflow forces the injected blood to flow through and past the right atrium, to be partly collected by the draining cannula in the inferior vena cava. A video showing the simulated streamlines in time is available in Additional file 2.

To give a more quantitative analysis we computed the recirculation fraction as the average percentage of oxygen, above the baseline, that flows out of the draining cannula, normalised by the ECMO flow. The idea is that, since venous blood contains only baseline oxygen (${70}\,\%$ saturation), all the oxygen above that level flowing out of the draining cannula must have come from the returning cannula.

**Figure 4:**
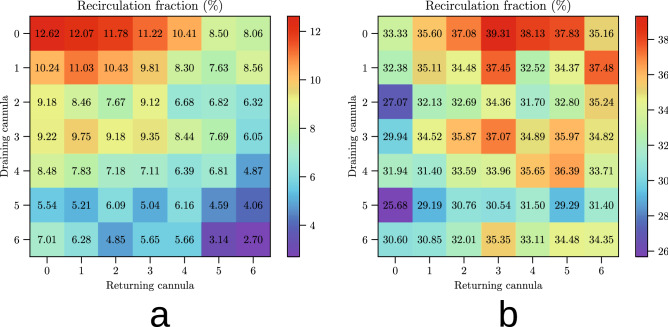
Recirculation charts for the two different ECMO flows. Oxygen recirculation for different cannula positions and the two considered ECMO flows. (**a**) 3 L/min. Mean: 7.66, standard deviation: 2.29. (**b**) $5\,\hbox {L}/\hbox {min}$. Mean: 33.57, standard deviation: 2.82. In the 3 L/min case a clear pattern emerges: when the cannulae are closer to each other, more recirculation occurs. In the $5\,\hbox {L}/\hbox {min}$, on the other hand, the blood comes in so fast that its behaviour is unaffected by the small changes in the cannulae’s positions. Regardless, recirculation is much more intense in the $5\,\hbox {L}/\hbox {min}$ case.

Figure 4 shows the values of the recirculation fraction for all our simulations. The $5\,\hbox {L}/\hbox {min}$ cases exhibit a lot of recirculation, more than ${30}\,\%$, while the 3 L/min cases only show about ${7}\,\%$ recirculation fraction. These values are similar to those obtained by^14^.

Furthermore, in the 3 L/min cases the positioning of the cannulae plays a minor role, showing that the most recirculation happens when the cannulae are closest to each other, while in the $5\,\hbox {L}/\hbox {min}$ case the cannula positions are irrelevant to the recirculation fraction.

### Oxygen availability

As a by-product of our simulations, we can inspect the predicted oxygen content in the right atrium given by the formula

3 $$\begin{aligned} C_aO_2 = {1.36}\frac{\mathrm{mL}}{\mathrm{g}} \cdot Hgb \cdot \frac{S_aO_2}{100} \end{aligned}$$

in which $Hgb = {12}\,{\mathrm{g/dL}}$ and $S_aO_2$ is the oxygen saturation.

**Figure 5:**
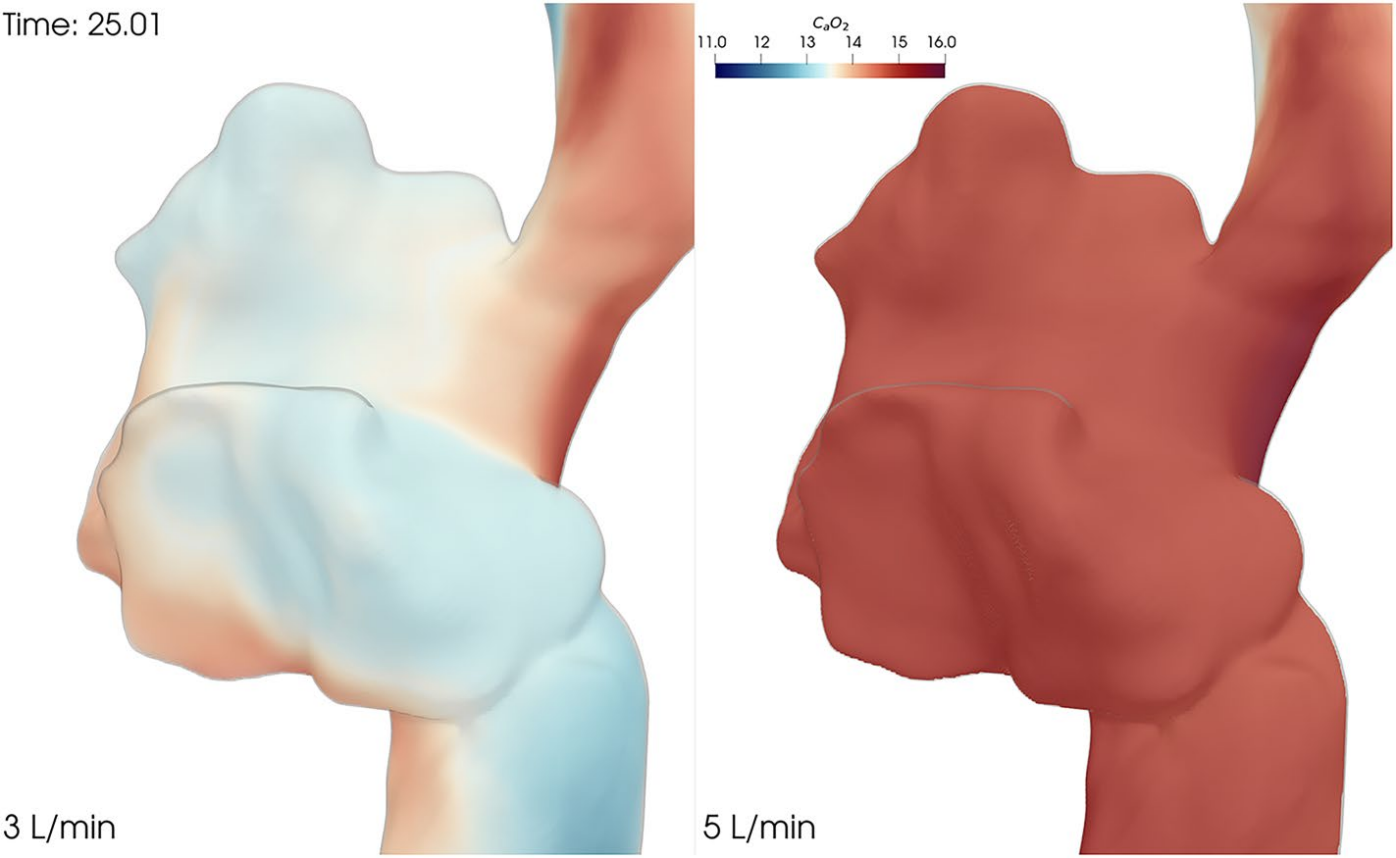
Volumetric representation of the oxygen content. Oxygen content ($C_aO_2$) in the right atrium for the 3 L/min (left) and $5\,\hbox {L}/\hbox {min}$ (right) cases after 25 s of simulation. This plot shows how an ECMO flow of 3 L/min, despite minimising recirculation, does not carry enough oxygen to adequately oxygenate the blood in the right atrium. With an ECMO flow of $5\,\hbox {L}/\hbox {min}$, on the other hand, a larger amount of oxygen accumulates in the right atrium, despite the increased recirculation stemming from a higher flow. Additional file 3 contains a video that clearly shows this phenomenon.

The difference is striking, as reported in Fig. 5, where we plot a volumetric representation of the oxygen content in the right atrium for configuration $(5, 5)$. In the $5\,\hbox {L}/\hbox {min}$ case, the oxygen content is much higher than in the 3 L/min case (a range of $[14.3, 15.6]$ versus a range of $[12.6, 15.3]$). Moreover, in both cases the highest oxygen content is where the flow from the returning cannula comes in but in the $5\,\hbox {L}/\hbox {min}$ case the whole atrium is well oxygenated while in the 3 L/min case the rest of the atrium has a pretty low oxygen content.

**Figure 6:**
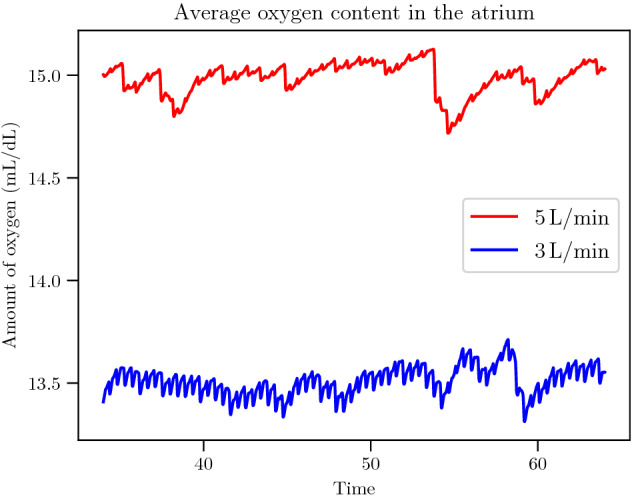
Total oxygen content in the right atrium during a sample simulation for the two ECMO flows we studied over the whole data-collection time interval. The plot shows that the difference in oxygen content in the atrium is sustained throughout the simulation and it is much bigger than the oscillations caused by the heart beats.

Additionally, we computed the average available oxygen in the right atrium as a function of time. This number directly expresses how much oxygen is available to the right ventricle when the Tricuspid valve opens. In Fig. 6 we compare the average oxygen content for configuration $(5, 5)$ for ECMO flows of 3 L/min and $5\,\hbox {L}/\hbox {min}$. The total amount of oxygen in the right atrium changes with every heart beat, following the open/close cycle of the Tricuspid valve. Regardless, there is significantly more oxygen available in the $5\,\hbox {L}/\hbox {min}$ case than in the 3 L/min at any given time. A video showing the simulated oxygen content in time is available in Additional file 3.

## Discussion

Our main finding is that the ECMO flow has a major impact on VV-ECMO efficacy, while the impact of the cannulae positioning is scarce. In addition, an important secondary outcome is that recirculation, more intense with a higher ECMO flow, is less detrimental to the procedure than previously thought.

### ECMO flow versus cannula positioning

The ECMO flow is more important than the fine tuning of the cannula positions. The saturation charts in Fig. 2 highlight the minimal impact of the cannula positions on the VV-ECMO outcome. In each of them, the values are pretty similar and there is not much difference between the best and the worst configuration. Figure 2a shows that the best configurations for an ECMO flow of 3 L/min are the ones with the returning cannula closest to the right atrium. One possible explanation is that, since the incoming oxygenated blood is slower, less of it flows into the inferior vena cava and more of it remains into the right atrium. This affects positively the effectiveness of the procedure. Nevertheless, the difference between the best and the worst cases is very small.

Figure 2b has a different gist: the best configurations for an ECMO flow of $5\,\hbox {L}/\hbox {min}$ are not necessarily the ones with the returning cannula at its closest and there seems to be no clear pattern. In this case, the blood flow is coming in much faster from the returning cannula and it significantly perturbs the flow of blood going into the Tricuspid valve, making the positioning of the cannulae less crucial.

The above observation is extremely relevant: when clinicians measure a low distal oxygenation, they can be tempted to try and adjust the position of the cannulae to increase the efficacy of ECMO, but once the cannulae are in position, moving them yields a major infection risk for the patient^17^. In light of the results reported in Fig. 2, fine-tuning the position of the cannulae can produce, in the best-case scenario, an improvement of barely ${2}\,\%$, which is hardly worth the risk of an infection.

### Effectiveness versus recirculation

Recirculation is commonly regarded as the major responsible for VV-ECMO’s limited effectiveness. In this direction, the recent work by^14^ showed that ECMO protocols with the highest cannula flow exhibit the most recirculation and are thus deemed less desirable. While it is true that minimising recirculation can be beneficial, this should not come at the cost of a smaller amount of oxygen available to the patient.

From our results we can conclude that a higher ECMO flow, despite yielding more blood recirculation, remains overall more effective than a lower one (Fig. 2). This apparent paradox can be resolved by thinking of what happens while the Tricuspid valve is closed. In those intervals some of the blood coming from the ECMO machine inevitably flows back into it through the draining cannula. The part of the oxygenated blood that does not recirculate remains in the right atrium, diffuses its oxygen and thus contributes to building a *reservoir* in the atrium itself. This oxygen accumulates and will be available the next time the Tricuspid valve opens.

Figures 5 and 6 back this remark and explain the overall better effectiveness of the $5\,\hbox {L}/\hbox {min}$ case (Fig. 2b) despite its higher recirculation (Fig. 3b).

### Potential implications of study results for medical care

The results presented thus far suggest a few key points that can be used to guide VV-ECMO administration.

First of all, the efficacy of current VV-ECMO protocols is intrinsically limited by the hemodynamics in the venae cavae and the right atrium, not by the specific protocol.

Secondly, fine-tuning the positions of the cannulae offers no significant potential benefits that balance the risk of generating an infection site in the patient.

Lastly, for current protocols and cannula designs, recirculation is a necessary evil stemming from the blood dynamics inside the patient.

### Limitations

Our study has some limitations. First, we only considered one patient and their individual geometry, and in principle one can expect that different anatomies may lead to slightly different results. This aspect deserves a more thorough investigation and will be the focus of our next study. Then, in our model we did not consider the compliance of the veins, an aspect of high complexity that requires a non negligible modelling and computational effort. Finally, as our focus was mainly on recirculation, we considered the Newtonian assumption for the blood to be sufficiently accurate, and the use of the Navier-Stokes equations to be justified. In our opinion, although deserving further investigation, we do not expect these limitations to have a significant impact on the global results of this study, as its main focus is on the effect of the cannulae positioning and the blood flow on the VV-ECMO efficacy. In particular, we expect individual geometries to have a minimal impact on the results, since the main anatomical feature favouring recirculation is the absence of any sort of barrier for the blood flowing from the superior towards the inferior vena cava. However, direct extrapolation of our results to the clinical setting should be made with caution.

## Conclusions

The most widely accepted explanation for the limited effectiveness of VV-ECMO is recirculation. We challenged this assumption on the belief that lower recirculation does not necessarily mean better oxygenation of the patient. What should really be optimised is the amount of oxygen that a given configuration delivers to the right ventricle.

Our study suggests that a higher ECMO flow should be preferred despite causing more recirculation. A lower flow actually yields a much lower oxygen availability in the system, which compromises to a larger extent the quality of ECMO.

Additionally, our results show that the exact positioning of the cannulae is not as relevant as previously thought, as it accounts for only a few percentage points in effectiveness.

The question of whether the best of both worlds—a lot of oxygen available to the heart and as little recirculation as possible—is achievable might find an answer in redesigning the cannulae in a way that favours blood delivery to the right ventricle.

## Supplementary Information

Supplementary Information 1.

Supplementary Information 2.

Supplementary Information 3.

## Data Availability

The datasets used and/or analysed during the current study are available from the corresponding author on reasonable request.

## Abbreviations

ARDS: Acute Respiratory-Distress Syndrome
VV-ECMO: Veno-Venous Extra-Corporeal Membrane Oxygenation
CT: Computer Tomography
