# Supplementary material for: Blood flow but not cannula positioning influences the efficacy of Veno-Venous ECMO therapy
Source: Sci Rep. 2022 Dec 5;12:20950. doi: 10.1038/s41598-022-23159-z (PMC9722702; doi:10.1038/s41598-022-23159-z)
Supplement: Supplementary file 1 — Supplementary Information 1. [file 41598_2022_23159_MOESM1_ESM.pdf]

RESEARCH

Computational model

Massimiliano Leoni[1*], Johannes Szasz[2], Jens Meier[2] and Luca Gerardo-Giorda[1,3]

*Correspondence: massimiliano.leoni@ricam.oeaw.ac.at
[1] Johann Radon Institute for Computational and Applied Mathematics, Linz, Austria
Full list of author information is available at the end of the article

Computational model

Cannula Design

The cannulae in our model geometry are loosely inspired by existing models manufactured by Maquet, in particular model BE-PAL 1923 for the returning cannula and model BE-PVL 2155 for the draining cannula. The former has an external diameter of 6.3 mm or 19 Fr while the latter has an external diameter of 7 mm or 21 Fr. Both cannulae have a thickness of 0.3 mm or 1 Fr.

Mathematical model

In this subsection we discuss the choice of equations that we use to describe ECMO. The blood in the venae cavae behaves as a regular incompressible Newtonian fluid, conserving mass and momentum, meaning its flow can be described by the incompressible Navier-Stokes equations

$$\begin{aligned} \rho(\partial_t \mathbf{u} + \mathbf{u} \cdot \nabla \mathbf{u}) &= \operatorname{div} \sigma(\mathbf{u}, p) \\ \operatorname{div} \mathbf{u} &= 0 \end{aligned} \tag{1}$$

where $\mathbf{u}$ is the velocity of the fluid , p its pressure and $\rho = 1050\,\mathrm{kg/m^3}$ its density [1]. The stress tensor $\sigma(\mathbf{u}, p) = 2\mu\varepsilon - pI$ is defined by the blood viscosity $\mu = 2.65 \times 10^{-3}\,\mathrm{kg\,m^{-1}\,s^{-1}}$ [1] and the symmetric velocity gradient $\varepsilon = \frac{\nabla \mathbf{u} + \nabla \mathbf{u}^T}{2}$, I being the identity matrix.

Equations (1) needs to be complemented with suitable initial and boundary conditions, the latter of which are notoriously the trickiest part of many modelling interventions in this field, and will be detailed in a later section.

We model oxygen as a passive scalar in the blood flow and its diffusion is accordingly described by a standard advection-diffusion equation

$$\partial_t c + \mathbf{u} \cdot \nabla c - k\Delta c = 0. \tag{2}$$

Without loss of generality, we consider the oxygen variable c to be between 0 and 1, with the role of measuring the difference in concentration between venous blood ($c = 0$) and fully oxygenated blood ($c = 1$). Saturation can be derived from the oxygen variable in the following way: for example, if the saturation level is 70 % for venous blood, and 100 % for fully oxygenated blood, then the conversion from the oxygen variable c to saturation S is the simple affine map

$$S = 70 + 30c \tag{3}$$

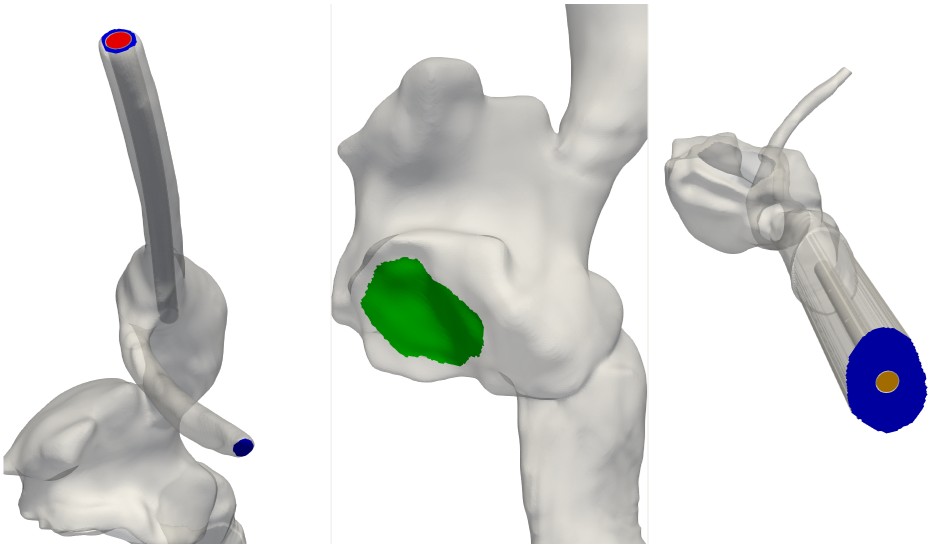

Figure 1: *Representation of the boundary conditions in our model.* These include the inlet of the returning cannula (red), the outlet of the draining cannula (orange), the inlets at the ends of the venae cavae (blue) and the outlet at the Tricuspid valve (green).

The choice of the diffusion coefficient k in Equation (2) deserves a remark. Unlike in usual passive-scalar diffusion, in which an inert substance is added to the fluid flow, in this case the oxygen is physically attached to the blood's red cells, which do not push it around or pass it to each other. The choice of Equation (2) is, however, still justified because, in the *continuum hypothesis* under which our model is valid, a small volume of blood contains a large number of red cells, hence the mixing of two volumes with different oxygen contents results in a volume which, on average, has an oxygen content in between the contents of the original volumes, meaning it still acts, in practice, as a diffusion process. For this reason we also pick $k = \frac{\mu}{\rho} = 2.52 \times 10^{-6}\,\mathrm{m}^2/\mathrm{s}$ with ρ and μ as in Equation (1), because oxygen will diffuse just like the same blood particles it is attached to.

Boundary conditions

Blood flow

While every solid wall is equipped with the standard no-slip boundary condition ($\mathbf{u} = \mathbf{0}$), suitable boundary conditions must be imposed, that appropriately describe the flow of blood entering the domain from the venae cavae and the returning cannula, and flowing out of the domain through the draining cannula and the Tricuspid valve. Figure 1 shows where the various boundaries are located.

Returning cannula For the returning cannula, which is an inlet, the blood flow rate is set on the ECMO machine. The injected blood flow being constant, the inflow velocity is a vector field with direction parallel to the cannula's axis and the characteristic parabolic profile of a steady-state flow in a pipe. If a value Q is given for the blood flow – typical values are $Q = 5\,\mathrm{L/min}$ or $Q = 3\,\mathrm{L/min}$ – then the

paraboloid's peak magnitude is $u_{in} = 2\frac{Q}{A\cos\vartheta}$ where A is the inlet surface area and ϑ is the angle between the inward normal to the inlet and the cannula's axis.

During a simulation's initialisation phase the cannula is switched off, in which case its boundary condition is a null velocity $\mathbf{u} = \mathbf{0}$.

Draining cannula The boundary condition for the draining cannula follows from a similar argument and we impose an outflow velocity boundary condition in an analogous manner.

During a simulation's initialisation phase the cannula is switched off, in which case its boundary condition is a null velocity $\mathbf{u} = \mathbf{0}$.

Pressure at the end of the venae cavae Since no information on the blood velocity was available at the sections at which our image segmentation stopped, we impose a pressure boundary condition. A zero-pressure condition would mean that the openings of the venae cavae lead into empty space or into an infinite prolongation that offers no resistance to backflow. As both would be unphysical conditions, we use a time dependent boundary condition instead, given by

$$\begin{cases} \dot{p}(t) = \alpha F(t) \\ p(0) = 0, \end{cases} \tag{4}$$

where $F(t) = \int_V \mathbf{u}(t)\cdot\mathbf{n}$ is the flow through inlet V at time t and $\alpha = 1 \times 10^7$ Pa/m^3 is a value tuned at the pre-processing stage. Equation (4) expresses the fact that a net outflow generates a steep rise in pressure, just like an actual backflow in a vein generates an opposing pressure due to the vein featuring venous valves that prevent backflow. The opposing pressure increases steeply but continuously, mimicking the fact that a vein, due to its high compliance, initially stretches a little bit to allow some backflow while generating an opposing pressure. Equation (4) is complemented with the constraint that the pressure should never drop below zero as we do not want the inlet to offer any resistance to the inflow of blood.

Pressure and velocity at the Tricuspid valve The Tricuspid valve is the most delicate part of the boundary as it drives the blood flow in our domain. Mimicking a real heart dynamics, the Tricuspid valve will periodically open and close, to let blood flow from the right atrium into the right ventricle. When closed, the Tricuspid valve is simply modelled as a zero-velocity boundary condition $\mathbf{u} = \mathbf{0}$.

The open Tricuspid valve is a different story. Lacking again any velocity information, we impose a time dependent pressure drop that follows the lines given by the *Wiggers diagram* shown in Figure 2, where the red and purple lines represent the pressure in the right ventricle and in the right atrium, respectively. The difference between these two values is the pressure drop that we impose in time to model an open Tricuspid valve.

This boundary condition needs to account for the fact that we are imposing a pressure difference between the Tricuspid and the inlets of the venae cavae, not between the Tricuspid and the atrium. The theory of the Navier-Stokes equations (1) ensures that, for laminar flow in a straight tube, the pressure drops linearly in the

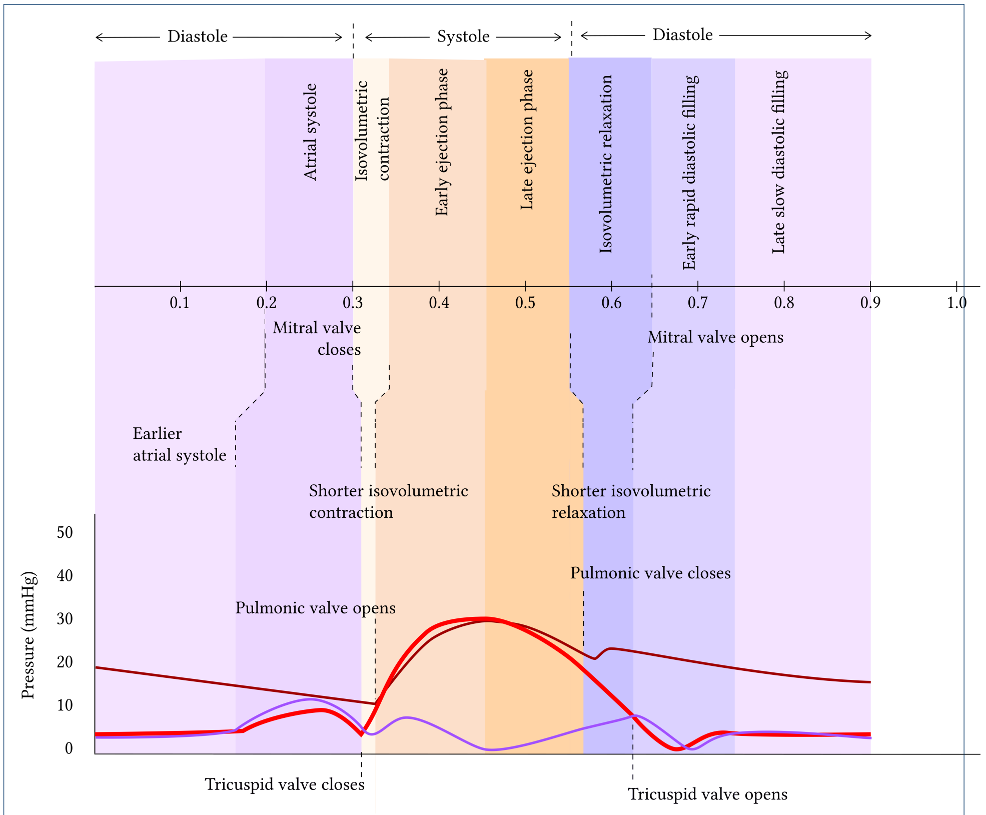

Figure 2: A Wiggers diagram. Reproduced with permission from https://derangedphysiology.com.

direction of the flow. As the venae cavae are roughly shaped like tubes, we scaled the pressure drop by a factor that is proportional to the distance from the inlets of the venae cavae.

Since, in our segmentation, the inlets of the Superior Vena Cava and the inlet of the Inferior Vena Cava were not at the same distance from the Tricuspid valve, we extruded the Inferior Vena Cava using CAD software until these three inlets actually do find themselves at the same distance from the Tricuspid valve, which in fact is approximately three times the distance from the Tricuspid valve to the centre of the right atrium. We thus scaled the pressure drop obtained from the Wiggers diagram by a factor of 3. During the post-processing of our simulation results we were able to verify that the pressure difference between the Tricuspid valve and the centre of the right atrium was around a third of the pressure drop, in agreement with the experimental data provided by the Wiggers diagram and thus validating our modelling approach. As additional validation, we verified that with this boundary condition our simulation predicts a physiological stroke volume.

Oxygen diffusion

On the inlets of the venae cavae, the oxygen variable is set to zero ($c = 0$) because the blood flowing into the domain from there is at venous oxygen level. The inlet of the returning cannula is set to $c = 1$ to model fully oxygenated blood. During

a simulation's initialisation phase the returning cannula is switched off, in which case its boundary condition is simply $c = 0$. Everywhere else in the domain we set $\partial_{\mathbf{n}} c = 0$ to state that oxygen will not diffuse across solid walls or outlets, but it can only be convected.

Physiological modelling

The Wiggers diagram in Figure 2 shows data for a heart beat that lasts 0.9 s, corresponding to a heart rate of 67 bpm, while experimental data report that ECMO patients have heart rates that vary in a range as big as $70-130$ bpm [2]. The pressure drop obtained from Figure 2 should thus be adapted to different heart rates.

The reason why a heart increases its rate is to pump more blood – hence more oxygen – into the circulatory system. For this reason, simply rescaling the pressure along the time axis will not do as, despite resulting in more beats, each beat will be shorter and the heart will be able to pump less blood than before. A sample numerical test promptly confirmed this claim.

An increased heart rate must yield an increased cardiac output; this much is self-evident. A trickier question to ask is how much bigger the cardiac output should be. Just like for the Wiggers diagram, there is plenty of data in the literature on what a physiological cardiac output (or, equivalently, stroke volume) is *in resting conditions*, meaning for a standard heart rate; data on the same quantities as a function of the heart rate is instead very scarce. Some of the little available data can be found in [3], where the authors report cardiac output values for various heart rates for humans at rest and during exercise.

We decided to model the effect of the heart rate on the pressure drop as a scaling factor – in addition to the aforementioned time scale, which of course we still feature. In order to fit this scaling factor as a function of the heart rate we resort again to the data reported in [2], which reports heart rates and cardiac outputs for eight patients affected by Acute Respiratory-Distress Syndrome.

The tuning works as follows. First we perform a linear regression of our eight data points to model the functional dependency of the cardiac output and the heart rate on the ECMO flow. We then look for a scaling factor, be it α, so that, setting in our software an ECMO flow of our choice and the corresponding heart rate as per the linear regression, we get the correct cardiac output – again, as per the linear regression above.

The linear regression data and results are reported in Figures 3 and 4. As these plots clearly show, the data is sparse and varies wildly. During our fitting procedure we ran, for each candidate value for α, 10 s of simulation (after 4 s of initialisation) and then we took the average stroke volume over that time interval; this is equivalent to estimating the cardiac output as the heart rate is fixed. We report that the stroke volumes had a high variance.

For an ECMO flow of 5 L/min we got a cardiac output of 3.81 L/min and a heart rate of 81 bpm, while for an ECMO flow of 3 L/min we got a cardiac output of 5.76 L/min and a heart rate of 134 bpm.

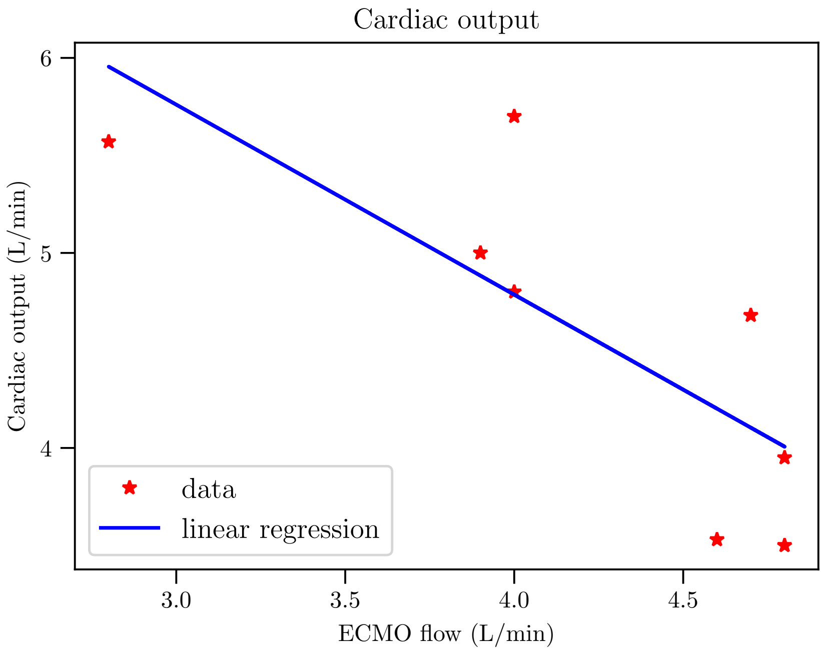

Figure 3: *Data and linear regression for the cardiac output as a function of the ECMO flow*. The regression line has equation $y = -0.97423x + 8.68303$.

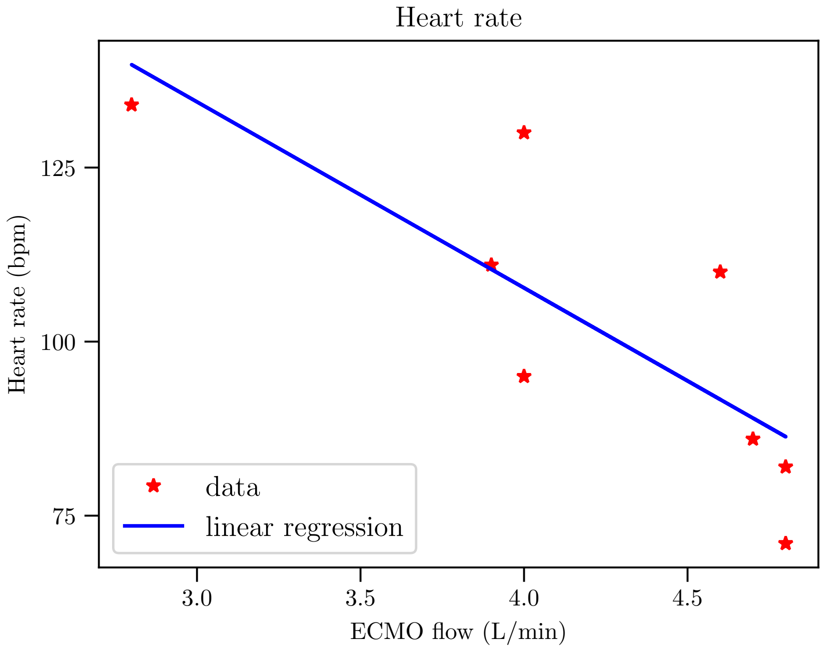

Figure 4: *Data and linear regression for the heart rate as a function of the ECMO flow*. The regression line has equation $y = -26.718x + 214.59$.

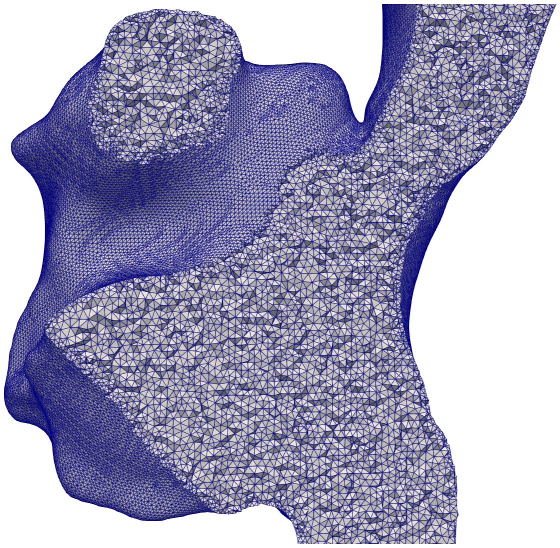

Figure 5: Detail of the mesh of the $(3,3)$ configuration. All our meshes have around 1.5 million cells.

Quantifying recirculation

The assessment of recirculation presented in the manuscript is based on the quantity

$$r = \frac{1}{t_f - t_i} \int_{t_i}^{t_f} \frac{1}{Q} \int_{C_{drain}} c\mathbf{u} \cdot \mathbf{n} \, \mathrm{d}x \, \mathrm{d}t \tag{5}$$

where C_{drain} is the outlet of the draining cannula, $Q = 3\,\mathrm{L/min}$ or $5\,\mathrm{L/min}$ is the ECMO flow and $[t_i, t_f]$ is the data recording interval. The quantity r represents the time averaged flux of oxygen through the draining cannula $\left(\int_{C_{drain}} c\mathbf{u} \cdot \mathbf{n} \, \mathrm{d}x\right)$, normalised by the ECMO flow Q.

Software and simulation details

We ran our simulation using in-house software based on FEniCS-X [4, 5, 6]. The meshes we prepared have around 1.5 million cells; Figure 5 shows the details of one of them. Our meshing process leverages the functionalities offered by Salome, Netgen [7] and Gmsh [8].

We checked mesh convergence for our quantity of interest, the blood saturation at the Tricuspid valve, by running the $(3,3)$ configuration on a finer mesh – around 2 million cells, with a reduction of h_{max} by a factor of three. The two meshes give values that differ by less than $2\,\%$.

We solve the Navier-Stokes equations with a fixed-point non-linear solver based on the technique presented in [9] with a relative tolerance of 0.01.

The oxygen diffusion equation is linear and we use a standard GMRES solver with Successive-Over-Relaxation preconditioner.

Author details

[1]Johann Radon Institute for Computational and Applied Mathematics, Linz, Austria. [2]Department of Anaesthesiology and Intensive Care, Kepler University Klinikum, Linz, Austria. [3]Institute for Mathematical Methods in Medicine and Data Based Modelling, Johannes Kepler University, Linz, Austria.

References

1. Hasgall, P.A., Di Gennaro, F., Baumgartner, C., Neufeld, E., Lloyd, B., Gosselin, M., Payne, D., Klingenböck, A., Kuster, N.: IT'IS Database for thermal and electromagnetic parameters of biological tissues, Version 4.1 (2022). doi:10.13099/VIP21000-04-1
2. Bond, O., Pozzebon, S., Franchi, F., Zama Cavicchi, F., Creteur, J., Vincent, J.-L., Taccone, F.S., Scolletta, S.: Comparison of estimation of cardiac output using an uncalibrated pulse contour method and echocardiography during veno-venous extracorporeal membrane oxygenation. Perfusion **35**(5), 397–401 (2020). doi:10.1177/0267659119883204
3. Chapman, C.B., Fisher, J.N., Sproule, B.J.: Behavior of stroke volume at rest and during exercise in human beings. The Journal of clinical investigation **39**(8), 1208–1213 (1960). doi:10.1172/JCI104136
4. M. S. Alnaes A. Logg, K.B.Ø.M.E.R., Wells, G.N.: Unified Form Language: A domain-specific language for weak formulations of partial differential equations. {ACM} Transactions on Mathematical Software **40** (2014). doi:10.1145/2566630
5. Scroggs, M.W., Baratta, I.A., Richardson, C.N., Wells, G.N.: Basix: a runtime finite element basis evaluation library. Journal of Open Source Software **7**(73), 3982 (2022). doi:10.21105/joss.03982
6. Scroggs, M.W., Dokken, J.S., Richardson, C.N., Wells, G.N.: Construction of arbitrary order finite element degree-of-freedom maps on polygonal and polyhedral cell meshes. ACM Transactions on Mathematical Software (2022). doi:10.1145/3524456
7. Schöberl, J.: NETGEN An advancing front 2D/3D-mesh generator based on abstract rules. Computing and Visualization in Science **1**(1), 41–52 (1997). doi:10.1007/s007910050004
8. Geuzaine, C., Remacle, J.-F.: Gmsh: A 3-D finite element mesh generator with built-in pre- and post-processing facilities. International Journal for Numerical Methods in Engineering **79**(11), 1309–1331 (2009). doi:10.1002/nme.2579
9. Houzeaux, G., Vázquez, M., Aubry, R., Cela, J.M.: A massively parallel fractional step solver for incompressible flows. Journal of Computational Physics **228**(17), 6316–6332 (2009). doi:10.1016/j.jcp.2009.05.019
